# Who Gets Cured? COVID-19 and Developing a Critical Medical Sociology and Anthropology of Cure

**DOI:** 10.3389/fsoc.2020.613548

**Published:** 2021-01-12

**Authors:** Maria Berghs

**Affiliations:** Allied Health Sciences, De Montfort University, Leicester, United Kingdom

**Keywords:** COVID-19, cure, sociology, anthropology, vaccine

## Introduction

In this opinion piece, I argue that a sociology and anthropology of cure is accelerated by various features of the scientific and social responses to the COVID-19 pandemic. I illustrate how the pandemic has made the general public rethink popular notions of “cure,” foregrounded ethical dilemmas and inequalities in who has access to “cures” and also revealed deep uncertainties correlated to a future where there is no such thing as cure anymore. Such developments in the pandemic response illustrate the need for a critical interdisciplinary agenda to interrogate the social, ethical, cultural, economic, political and technological innovations of cures nationally and internationally.

The race for a vaccine for the SARS-CoV-2 virus that causes COVID-19 illustrated the urgency to find a cure during a pandemic but also deep anxieties, as the general public realizes they have to leave behind absolutes of “cure” and deal with uncertainties of who now gets cured? In medical sociological and anthropological literature, absolutes of cure have long been criticized in research, amongst others, focusing on changing ideas of: inequalities in who becomes incurable or curable, for example, during the HIV/AIDS epidemic (Schoepf, 2001; Nguyen, 2010), inclusion in clinical trials (Petryna, 2009), or due to genomic advancements (Inhorn and Wentzell, 2012); environmental, lifestyle and embodied (epigenetic) risks which have reconceptualised understandings of nature and nurture (Kavanagh and Broom, 1998; Lock, 2013; Gale et al., 2016), as “situated biologies” mean rethinking notion of bounded bodies in favor of how biology is affected by environment (Niewöhner and Lock, 2018); expectations and hopes of new biotechnologies and artificial intelligence that bring to the fore the way in which scientific advancements can politically shape subjectivities, temporality, emotions and care (Brown and Michael, 2003; Brown, 2005; van der Niet and Bleakley, 2020); “promissory futures” of biomedical and scientific innovations, such as in the field of regenerative medicine, become correlated to neoliberal policy-making and economic investments (Brown et al., 2006; Selin, 2008; Morrison, 2012); novelty, for instance in epigenetics, becomes socially constructed (Pickersgill, 2020); clinical forecasting is relationally imbedded and negotiated in clinical practices (Timmermans and Stivers, 2018); and dealing with uncertainty of conditions with no cure, where instead experiments become perilous options for patients (Fox, 2020). The above is just a sliver of the breadth and depth of knowledge built on a continuum of cures, but the very notion itself of “a cure” and how the concept is changing, is never explicitly questioned as such.

Yet, as illustrated, just as comprehension of COVID-19 is marked by social understandings of inequalities in infection, environment, prevention and intervention (Lupton and Willis, 2020; Trout and Kleinman, 2020), knowledge of cure is socially and culturally informed too. Public health pandemic responses to COVID-19 have focused on scaling up disease prevention and control efforts, public health information, laboratory systems and development of private and public partnerships to develop diagnostics, therapies, antiretrovirals and vaccines. Furthermore, critical social commentaries have been noted in terms of ethics of access to: care; life-saving equipment like ventilators; therapies (e.g., remdesivir); vaccines; as well as debunking the idea of recovery and immunity.

Presently, with hopeful vaccines on the horizon (Horton, 2020), a “critical bioethics of cure” is developing, informed by differing ethical norms and values in society, associated to who gets access to vaccines and how they will be allocated (see, Emanuel et al., 2020). For example, in the United Kingdom (UK), the disability community has warned of overt discrimination in lack of ethical inclusion in pandemic preparedness and response (Armitage and Nellums, 2020), “ableism” (Campbell, 2009) of foregrounding of able body in withholding, triage or rationing of care as cure, as well as warning of “social” deaths in our disablist language use, and real deaths in revoking of rights in health and social care policies (Abrams and Abbott, 2020; Tidball et al., 2020).

Disability studies researchers, while long critical of the medical model and curative imperative (Clare, 2017), are pointing to an unethical “curation” or “social sorting” (Grover and Piggott, 2010) in how the able body now gets protection against an infection, access to critical care, therapies and vaccines (Scully, 2020), according to a new curative “imperative of health” (Lupton, 1995) or distributed “logic” of cure (Mol, 2008). The logic of cure describes how an “imperative of cure” becomes normalized in our social and cultural lives and is increasingly commodified but not distributed equally nor a choice. Neoliberalism and promises of late modernity have been incorporated in such a logic of cure, in terms of a “biopolitics of cure” in how patients, doctors, researchers, pharmaceutical companies and financial investors create momentum around specific infectious diseases, genetic disorders, chronic or neurological conditions and now in its acceleration for the general population during the COVID-19 pandemic.

## Rethinking Cure

COVID-19 elicits a variety of human immune responses (e.g., acute, chronic, mild, and also uncertain recovery) that we do not yet understand, in both people who are seemingly healthy or have pre-existing conditions. We know that that certain sections of the population (e.g., linked to structural inequalities, ill-health, co-morbidities, age, disabilities and biology) are at greater risk from COVID-19 (Bentley, 2020). People who have COVID-19 can also be asymptomatic carriers (see Gandhi et al., 2020), as well as possibly get reinfected after recovery, further complicating our ideas of symptoms and signs, as well as clinical and social understandings of how the virus spreads. While patients recover, it does not seem as if immunity is always long-term or sustained, calling into question ideas like giving survivors “immunity passports” (Andersson et al., 2020). Similarly, “vaccine certificates,” “identification cards” or “vaccine passports,” which while clinically and practically useful, could open up the door to legal, ethical, and social issues, such as discrimination of those without vaccinations (Phelan, 2020).

Further complicating notions of immunity and long-term cure, is that COVID-19 also has “impairment effects” (Thomas, 2007) in creation of impairments (e.g., organs), affects senses (e.g., smell) and emotions (e.g., post-traumatic stress disorder) with physical and psycho-social long-term rehabilitative needs (Halpin et al., 2020; Mandal et al., 2020). As such, Greenhalgh et al. (2020) have noted the emergence of patients who have survived COVID-19 but whose clinical and mental health recovery is slow and long, noting that these patients are termed “long haulers.” New “biosocial” categories (Rabinow, 1996; Rose, 2009) of patients are thus emerging around social identities of uncertain survivorship from COVID-19, as we discover more about how COVID-19 affects people (Kingstone et al., 2020; Ladds et al., 2020; Miyake and Martin, 2020; Philip et al., 2020).

While a sociology of diagnosis (Nettleton, 2006; Jutel, 2009) can be helpful to comprehend patient needs for a medical diagnosis, people with long COVID-19 struggle with the physical and mental health uncertainties of recovery and realization that there may only be a partial survivorship or indeterminate forms of cure (see Ladds et al., 2020). Similarly, there is no certain prognosis or forecasting that can be made about the future of how recovery from COVID-19 survivorship will unfold alongside other conditions, and this influences treatment options and experiences of primary care (Kingstone et al., 2020). The current medical emphasis is still on comprehension of the embodiment of curative processes and examining prognosis, treatment and responses to therapies, rehabilitation, mental health support and how survivor experiences can become linked to prevention efforts.

Attending to risks of COVID-19 and mitigating those through policies such as lockdowns, means the indirect effects of who does not get access to diagnosis, therapies and curative promises in the NHS and whose health and impairment is ignored, has been neglected in research. As have the social realities of the thousands of people who have been told to shield because they are severely clinically vulnerable. We do not yet understand the psychological and social impacts on this population group of long-term shielding and messages of “vulnerability” directed toward them. They and their loved ones have had to deal with the idea that survivorship from COVID-19 may not be a possibility for them, as well as having heightened levels of risk to negotiate. What has been the physical and psychological impact of such heightened risk work of staying well? There will also be people within this group that will survive COVID-19 but we don't know if there is a continuum of mild, moderate and severe short or long-term effects, nor if there are more curative possibilities that will be created in the future?

## Promissory or Unequal Futures?

Promising candidate vaccines and research initiatives have raised local and global public hopes and expectations of promissory futures (Brown et al., 2006) of living COVID-19 free and returning to a normal life. However, these hopes have been tempered by clinicians, academics, scientists, and philanthropists involved in pandemic efforts noting the need for more long-term research about effectiveness of vaccines (Horton, 2020). For example, while the Pfizer/BioNTech, Sinovac and Moderna mRNA candidate vaccines appear to offer initial effectiveness, results have yet to be published scientifically and appraised by national regulatory bodies (Horton, 2020), although the UK has approved the Pfizer vaccine. Similarly, while the Oxford/AstraZeneca candidate vaccine has also reported high rates of efficacy, dose errors meant more testing was needed. In addition, UK's Royal Society The DELVE Initiative (2020) have warned difficult medical, political, ethical, economic, cultural, gendered and social questions remain about vaccinations, such as equitable allocation and their long-term effectiveness.

The UK's policy responses have been steeped in self-interested nationalism, for instance, by not engaging in European public-private partnerships or research platforms and insisting on British development of UK vaccine (Sharpe et al., 2020). Likewise, the UK's public health arguments and pandemic responses often emphasize individual civic responsibilities for the common good (e.g., to get tested or vaccinated) rather than broader structural arguments about “affordability, resource allocations and accountability” that the government is responsible for (Forman and Kohler, 2020). Very little policy attention has also been paid to the need to rebuild trust nationally and internationally in government and health services, for instance, due to impact of COVID-19 on ethnic minority communities and health care professionals, who are also most affected by health inequalities, structural racism and history of medical mistreatment (Bentley, 2020). Surveys have reported that those most affected by COVID-19, are more likely to report fears and less likely to want to be the first ones allocated to participate in vaccination efforts (see Thorneloe et al., 2020). This also raises further questions about accessibility of vaccines, if there will be multiple offers of vaccinations and if people can choose if they want to be vaccinated or not, and with which vaccine? What types of choices will people have? Will those be constrained by nationalism? This remains to be seen as the Pfizer vaccination begins and the UK heralds itself as being the first in the world to begin a mass vaccination campaign to protect against COVID-19.

While taking part in scientific research and trials for vaccines has undoubtly opened new transnational ideas of curative citizenship (Rose, 2009), in the sense of acting for the common global good to find a cure, access to vaccines seems bound to citizenship and not to ideas of social justice, racial equity or biological or social needs. This is reinforced by therapeutic and vaccine hoarding that certain nations in the Global North have been engaging in. For example, Trump trying to gain exclusive access to a vaccine for the United States by buying up stocks for national interests (see Dyer, 2020), rather than fulfilling the potential and promises of collaborative academic and private-public partnerships for global equity, solidarity and rights to health (Forman and Kohler, 2020).

It's important to interrogate how this could have happened? While philanthropic organizations such as the Bill and Melinda Gates Foundation and the Wellcome Trust have been involved in setting up collaborative research platforms for cures and setting curative agendas for equity, the realities of pandemic preparedness mean that transnational partnerships can be quashed for national interests. This points to the need to interrogate how “cure” functions and for which political and economic interests. Philanthropic organizations have also paid less attention to the possible ramifications of the narrow development for cures without correlated investments in care and social equity. By way of illustration, what is the point of developing a cure for a neglected tropical disease, if you can still get seriously ill because the basics of healthcare are neglected (Berghs et al., 2020). Are there barriers in ethically interrogating or calling into account such inequalities in curative development? As such, this points to the importance of questioning definitions of cures, trajectories of their development and by whom curative agendas get set during pandemics.

## A Research Agenda?

To critically interrogate who gets cured, I argue that a new interdisciplinary research agenda is needed that builds on the theoretical tools that we have, to develop a medical sociology and anthropology of cure. Kavanagh and Broom (1998) emphasized that if you wanted to understand intersection between environmental and embodied risks, it was important to work together with people at “risk” to formulate new languages for changed norms and values, as well as approaches to novel environmental and socially embodied understandings. Similarly, a bioethics of cure could be an empirical-ethical theory that could develop from the experiential knowledge of patients with COVID-19 undergoing diagnosis, therapies and experiencing differing forms of cure (Caron-Flinterman et al., 2005) or undertaking differing forms of “curative labor” (Cooper and Waldby, 2014). Yet, we are all currently socially and culturally engaging with diverse materialities of cure in various settings.

There is an emotional and physical “curative labor” involved in gaining expertise on immunity or using techonologies to stay “well,” keeping others healthy and negotiating curative risks of COVID-19 that we are all involved with. There are also people who will be identified as having more potentialities or probabilities to be cured and others that refuse cure. Likewise, many people are living in fear, shielding or bereaved and dealing with loss of curative hope and inequalities of cures. In a sociological sense, we have all gone through a biological disruption (Bury, 1982) and are dealing with the reality of “no cure” which has profoundly altered our worlds.

Hacking (2006) stated that people would socially organize around new types of genetic risks, but I argue that new forms of identity are emerging, not only in terms of pandemic risks and cures but concerning novel immunotherapeutic and curative risks of anti-microbial resistance, potentials and dashed hopes which are unsettling epistemologies and ontologies of how we understand biology, identity, embodiment and environment. We have the tools to socially frame this new world together with the people most affected, not only for the next pandemic but also with respect to novel developments in cure. We have to engage in interdisciplinary work with epidemiology, public health, science and technology studies, economics, disability studies, psychology, politics, ethics, law and so on, to understand the impact of the search, development, potentials and realities of agendas for accelerated searches for cures and their impacts. We need to locate “cure” in pandemic preparedness but also wider scientific debates and biomedical and technological developments. What could “cure” now mean?

## A New Sociology and Anthropology of Cure Should:

1. Investigate how conceptions of cure politically change during pandemic responses and as a part of national and international agendas of technological innovation. Why does methodological nationalism but also the harsh policing of national borders, for instance, happen during acerated curative searches?;
2. Critically examine and question the local and global inequalities in who gains access to care as cure and the (bio) ethical, social, financial, political, cultural and historical decisions that underpin such access. For example, who is going to gain first access to a vaccine globally and what are the underpinnings of such policy decisions? What is curative nationalism?;
3. Understand the expectations, emotions, expertise and embodied experiences of what it means to undergo cure as patient, make sense of limitations of cure and/or lack of cure. For instance, how does it feel to survive COVID-19 and realize that recovery may only be partial? What psychological care and social support is needed?;
4. Frame the local realities of cure against broader transnational activism and global debates linked to research for cures by focusing on how biological data is interpreted through kinship, gender, ethnicity and disability. What does it mean to be part of an accelerated search for a cure, such as a patient in a vaccine trial, and how do people understand their involvement and how their biological data will be used?;
5. Map what needs exist for patients and their families, with respect to understanding new scientific developments linked to diagnostics, therapies, vaccines and cures. What information is needed by families who undergo latest curative interventions, such as gene editing or stem cell donations? How do they understand their curative trajectory and identity post-cure? Does a biopolitics of cure develop?;
6. Chart what future impact a growing field of cures would have on health and social care services for patients where treatments are not an option, as well as disability activism and advocacy. How does cure become linked to time and notions of “normality”? Does a focus on cure lead to ableism in society and increase the imperative of health? Does this increase curative stigma?;
7. Understand the norms and values of scientific involvement in diagnosis, therapies and vaccines for cures and if those are reflected by professionals working in development and financing of cures. Does a research scientist view their work as “curing”? Is that the same as the people who finance the cures or big philanthropic organizations?;
8. Learn what impacts cures have when viewed alongside existing inequalities that affect patients in local and global contexts. Are there unintended impacts of cure? What role does artificial intelligence have in development of cures for patients or identification of patients who might need cures? How is accessibility to both testing for need of cure and cure itself ensured ethically, in for example, personalized medicine?;
9. Investigate how information in a local perspective on cure connects to broader transnational and transgenerational debates to explore the ethical, economic, political, legal and historical implications of cures and searches for vaccines. Are new developments for cures connected to previous histories and pandemics, for example, Ebola? How do people make sense of those pasts in the present? Why don't we interrogate a logic of cure the same way we do care?

## Author Contributions

The author confirms being the sole contributor of this work and has approved it for publication.

## Conflict of Interest

The author declares that the research was conducted in the absence of any commercial or financial relationships that could be construed as a potential conflict of interest.
